# A Scoping Review of Factors Associated With the Mental Health of Young People Who Have “Aged Out” of the Child Welfare System

**DOI:** 10.1177/15248380231196107

**Published:** 2023-09-30

**Authors:** Alice R. Phillips, Sarah L. Halligan, Iris Lavi, John A. A. Macleod, Susan Robinson, David Wilkins, Rachel M. Hiller

**Affiliations:** 1 University of Bath, UK; 2 The National Institute for Health Research Applied Research Collaboration West (NIHR ARC West) at University Hospitals Bristol and Weston NHS Foundation Trust, UK; 3 The University of Bristol, UK; 4 Cardiff University, UK; 5 University College London, UK; 6 Anna Freud Centre for Children and Families, London, UK

**Keywords:** scoping review, qualitative, quantitative, mental health, foster care, child welfare

## Abstract

Young people who grow up in care and then exit care around the age of 18 (care-leavers) are a particularly vulnerable group, at increased risk for mental health problems even relative to other care-experienced groups. Yet, little is understood about the factors underpinning this association. We used scoping review methods to synthesize the quantitative and qualitative literature on factors that are associated with mental health problems for care-leavers. Following rigorous methods, we systematically searched three scientific databases spanning psychology and social care and identified 23 peer-reviewed studies for inclusion. This review highlights the heterogeneity of this research, in terms of methodology and topics investigated. Topics included are as follows: pre-care maltreatment, care-related experiences, psychological factors (emotion regulation), social support, education, and adult functioning (e.g., housing, finances, employment). We found mixed and inconsistent findings across research studies. The strongest evidence-base is around the influence of social support upon the mental health of recent care-leavers, though methodological problems are discussed. The field benefits from several large-scale observational and longitudinal research studies. However, there is an over-reliance upon retrospective reporting, and the use of unvalidated measures is common. It is apparent that there are significant gaps in our current understanding of the mental health of care-leavers, in particular around modifiable factors. We discuss potential directions for future empirical research, both in terms of methodology and factors investigated.

## Introduction

The most common reasons a young person is removed from the care of their biological parent and placed in the child welfare system are abuse, neglect, or family dysfunction, or a combination of these (Cameron et al., 2018). Once in care instability can continue, including separation from siblings and regular changes in caregivers/placement providers (Hiller & St. Clair, 2018; Hiller et al., 2023; Rubin et al., 2004). It is also the case that young people in care are at high risk of revictimization and exploitation, particularly during adolescence (Euser et al., 2016; Katz et al., 2020). There is well-documented evidence of the high rates of mental health difficulties experienced by young people in care. An international systematic review found that between 32% and 80% of all young people in care meet the criteria for a mental health problem, compared with around 18% in the general population (Steel et al., 2014). Conduct disorder, major depressive disorder, and posttraumatic stress disorder (PTSD) appear to be the most common difficulties experienced by care-experienced people across their lifetime (Engler et al., 2022). Mental health problems are more likely to be comorbid, and suicidal ideation and behavior were also much higher (Engler et al., 2022). Heightened risk of psychological difficulties continues into adulthood, with a meta-analysis showing that care-experienced adults have a lifetime prevalence rate of 30% for any mental health disorder, compared to just 18% in the general population (Seker et al., 2021).

Internationally, there is an increasing number of young people entering care in later childhood. Young people who enter care during adolescence are the most likely to remain in care until they reach adulthood and “age out” (Clarke & Penington, 2021). There is growing evidence of the significant unmet needs of young people who “age out” of care (Havlicek et al., 2013). Oftentimes, this group is referred to as “care-leavers” and are a subgroup of the wider “care-experienced” population. Care-experienced refers to anyone for whom the state gained legal guardianship, oftentimes through children’s social services. Although the exact definition of a “care-leaver” differs between countries, in this review care-leavers are people who were placed under the care of children’s social services (i.e., the state gained legal guardianship), but they also remained in care until they reached the maximum age permitted (usually around 18 years old). Some countries provide mechanisms for further support beyond this age (e.g., parts of the United States and the UK), but there is little evidence on whether this support is accessed by young people who exit care (Strahl et al., 2021). For example, only 31% of 19- and 20-year-old care-leavers “stayed put” with their foster carers during 2020–2021 in the United Kingdom (Department of Education, 2021).

Despite differences in welfare systems across countries, it is the case that care-leavers’ outcomes are consistently poor in terms of disability, education, employment, income, and housing when compared to the broader care-experienced population (Berlin et al., 2011; Cameron et al., 2018). There is some evidence that care-leavers may have a more disrupted experience within the care system, indicated by greater placement instability and more instances of running away (Shook et al., 2013). In adulthood, care-leavers are at also at increased risk for mental health problems, substance abuse difficulties, and death by suicide than the broader care-experienced population (Hamilton et al., 2015; Shook et al., 2013). Unique to care-leavers is the accelerated transition into independence, at a much younger age than the general population (Baker, 2017). Support for the transition out of care differs immensely across and within countries (Strahl et al., 2021). In the United Kingdom, where care-leavers can access support until around the age of 25, qualitative research suggests mixed experiences of this support. Some young people enjoy their new independence and benefit from the post-care support offered, while others feel abandoned, powerless, and struggling with instability (Butterworth et al., 2017). Evidence suggests that the process of leaving care itself increases vulnerability among care-leavers, with increased chances of homelessness, and worsening of extant mental health problems (Dworsky et al., 2013; Stein & Dumaret, 2011). Despite the evidence that care-leavers are vulnerable to poor mental health outcomes even relative to other care-experienced groups, a clear understanding of the factors associated with this is lacking. Such evidence is crucial for understanding where resources might be best placed to intervene to either prevent or minimize poor psychological outcomes. Part of the challenge in factors associated with outcomes in this group is synthesizing and interpreting the existing evidence. The literature is spread across multiple disciplines. Although much of our current understanding of the needs of care-leavers is informed by large-scale research within the field of social work (e.g., Courtney et al., 2001; Fernandez et al., 2017; Pecora et al., 2006), relevant work is also found in psychology, psychiatry, and broader allied health disciplines.

To address our limited understanding in this area, we conducted a scoping review of the literature on broadly defined “factors” which are related to the mental health of care-leavers. Given the definition of care-leavers differs between regions, this review specifically focused on the mental health of any person who legally “aged out” of care (generally between 16 and 18 years old). Scoping reviews aim to provide insight into where the literature currently stands; therefore, “factors” was broadly defined to include any research capturing psychological-, social-, or system-level factors associated with mental health outcomes in this group. Research could be qualitative or quantitative, across any country, and from any discipline. As this is a rapidly developing research field, the primary aim of our scoping review was to summarize what we currently know about associations with mental health for care-leavers and highlight where the gaps remain.

## Method

We aimed to map research investigating the mental health of care-leavers, and the factors are related to this, using scoping review methods. We pre-published our scoping review protocol on the Open Science Framework (see: Phillips et al., 2022). Since the original protocol was published, some key alterations were made which are summarized below. We conducted our review using Covidence (Veritas Health Innovation, 2021), and standard procedures recommended for scoping reviews (Peters, 2017; Peters et al., 2015), and reporting according to PRISMA guidelines (Page et al., 2021).

### Inclusion Criteria

1. Peer-reviewed primary research studies using qualitative or quantitative methods
2. Published at any time and written in English
3. Includes analyses focused specifically on care-leavers, defined as any person who was under state care when they crossed the age of eligibility for being under state care (i.e., they “aged out” of care)
4. Includes qualitative investigation of mental health, and/or quantitative measurement of mental health using self-report or other-report (e.g., teacher, caregiver), via interview or questionnaires, or using records to identify the presence of mental health problems (e.g., health records, social care records). Articles that combine mental health alongside other outcomes in analyses were included (e.g., employment and mental health outcomes).
5. Investigates at least one broadly defined potential influencing factor in relation to mental health. For example, factors could include any external service factors (e.g., age of exit from care), individual psychological factors (e.g., self-assessed preparedness to exit care), or social processes (e.g., perceived social support).

### Search Strategy

We used a combination of search terms intended to be inclusive of all potential terminology referring to mental health and care-leavers. These were adapted for the functionality of the databases used and were developed with guidance from a university subject-specific librarian. Broadly, we searched using terms related to being a care-leaver (e.g., care-leaver OR aged out OR foster alumni) and terms related to mental health (e.g., mental health OR psychopathology OR emotional problem). Full search terms can be found in Supplemental Material A. These search terms were run within the databases Web of Science Core Collection, APA PsycNet, and Scopus. References of included papers were hand-screened for any overlooked papers not identified in the initial search.

### Screening and Data Extraction

**Figure 1.**
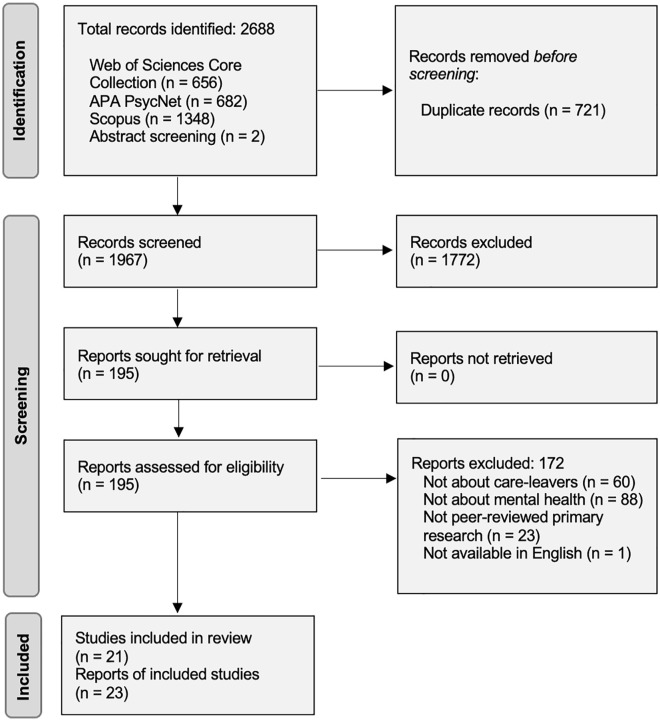
PRISMA flow diagram.

Searches identified 2,688 potentially relevant articles, which were reduced to 1,967 once duplicates were removed (see Figure 1 for a PRISMA diagram). The titles and abstracts of these 1,967 articles were screened against pre-defined inclusion criteria (see above). Reviewer one (lead author) screened all potentially relevant articles independently (*N* = 1,967), and then half of them were independently screened by another screener (*n* = 984). Reviewers achieved a 98% agreement (agreement was reached for 963 out of 984 articles). Discrepancies were resolved through discussion in relation to inclusion and exclusion criteria, moving articles to full-text screening any uncertainty remained. In total, 195 articles were moved to the full-text screening stage.

Two reviewers (initials removed for blinding) each screened half of the full-text articles for inclusion/exclusion. Ineligible sources were assigned a code indicating the reason for exclusion. Where there was insufficient or missing information within the full text, we contacted the manuscript authors for further information. As a check of screening accuracy, 20% of sources were independently screened by both reviewers, with 100% agreement. Of the 195 potentially relevant articles identified for full-text review, 23 were included in this scoping review (see PRIMSA diagram in Figure 1 for exclusions).

Sources were split evenly between two reviewers (ARP and SR), and extraction was conducted using a charting form. 20% of extraction completed by ARP was checked by SR.

### Changes From Pre-Published Protocol

We made the following edits to the pre-published protocol. We originally proposed to look at the evidence for the “transition” out of care. However, the pilot showed this initial conceptualization was too narrow. Thus, the review was broadened to focus on care-leavers, rather than specifying the focus on their transition out of care. Second, we changed our abstract screening process to be more thorough in terms of the percentage of articles screened as part of inter-rater reliability. Finally, we had originally proposed to scope the international gray literature. However, it proved unfeasible to scope and review the gray literature worldwide by including, for example, reports by country-specific public bodies, and charitable organizations. Due to concerns about the potential bias that could be introduced by a selective approach to the gray literature, compared to the systematic nature of the academic review, we ultimately decided to focus only on the academic literature.

## Results

### Descriptives

A total of 23 studies met our inclusion criteria. As shown in Table 1, most were published within the past decade (*n* = 16), with the earliest published in 2006. All studies were conducted in developed western countries; almost half were from the United States (*n* = 11), followed by a small number of studies from Australia (*n* = 2), Canada (*n* = 2), Israel (*n* = 2), Spain (*n* = 1), Sweden (*n* = 1), and the United Kingdom (*n* = 3).

**Table 1.** Overview of Included Research Studies.

Author(s)	Country	Data	Design	Sample	Outcome Measure(s)
Berlin et al. (2011)	Sweden	Quantitative, SDA of administrative records	Longitudinal, administrative record linkage study	Care-leavers (*n* = 5,224) In-home interventions (*n* = 6,455) National adoptees (*n* = 1,206) Non-involved groups (*n* = 900,322)	Suicide: admitted to hospital because of attempted suicide or suspected after 20th birthday using national administrative records
Cashmore and Paxman (2006)	Australia	Quantitative	Longitudinal, collecting data 3 months before exiting care, 3 months after, 12 months after, and 5 years after	Care-leavers (*N* = 47)	Adult functioning: using self-report interviews, participants were given a summed score ranging from 0 to 7 based upon “achievements” in employment (employed/education); stability of housing (never homeless); education (completed secondary school); substance use (no self-reported problems with alcohol or drugs); mental health (no reported depression or suicide ideation); criminal behavior (no admissions/self-reports, no convictions); and relationships (not violent or troublesome)
Dixon (2008)	UK	Quantitative	Longitudinal, collecting data 3 months before exiting care and again 12 months afterward	Care-leavers (*N* = 106)	Psychological distress: current symptoms, using the GHQ-12
Fowler et al. (2011)	USA	Quantitative	Longitudinal, collecting data every 3 months after exiting care, until 2 years later	Care-leavers (*N* = 265)	Psychological distress: current symptoms, using the BSI
Goldstein et al. (2013)	Canada	Quantitative	Cross-sectional	Care-leavers (*N* = 93)	Depression: 10-item CES-D
Grey et al. (2015)	USA	Quantitative	Cross-sectional	Care-leavers (*N* = 172)	Depression: current symptoms, using the depression subscale of the TSC-40 Anxiety: anxiety subscale of the TSC-40
Gullo et al. (2021)	Spain	Quantitative	Cross-sectional	Care-leavers who were unaccompanied migrants (*n* = 68) Other care-leavers (*n* = 73)	Mental health treatment access, suicidal ideation and attempts: past year prevalence, using a semi-structured interview
Kessler et al. (2008)	USA	Quantitative, mixed sources	Cross-sectional	Casey program care-leavers (*n* = 111) Other care-leavers (*n* = 368)	Mental health diagnosis: meet criteria within the past 12 months, using version 3.0 of the World Health Organization Composite International Diagnostic Interview
McCauley (2021)	USA	Quantitative, SDA of administrative records	Longitudinal, administrative record linkage study	Care-leavers (*N* = 5,221)	Substance abuse: administrative health records were used to find indicators that participants struggle with substance abuse
Melkman (2017)	Israel	Quantitative	Cross-sectional	Care-leavers (*n* = 354)	Psychological distress: The BSI-18
Miller et al. (2017)	USA	Quantitative, SDA of existing study data*	Longitudinal, collecting data between ages 17 and 19 years	Care-leavers (*n* = 404)	Mental health diagnosis: meet criteria for depression, PTSD, or anti-social personality disorder within the past 12 months, using the Diagnostic Interview Schedule-Version IV Substance use disorders: meet criteria within the past year, using the CASI-A Current depressive symptoms: depression outcomes module
Munson et al. (2015)	USA	Qualitative	Cross-sectional	Care-leavers with mental health problems (*N* = 59)	Not applicable
Narendorf and McMillen (2010)	USA	Quantitative, SDA of existing study data*	Longitudinal	Care-leavers (*N* = 325)	Substance use disorder: met criteria for current diagnosis using Diagnostic Interview Schedule, Version IV
Newton et al. (2017)	UK	Qualitative	Cross-sectional	Care-leavers (*N* = 11)	Not applicable
Prince et al. (2019)	USA	Quantitative, mixed sources	Longitudinal, data collected at ages 17 and 19	Care-leavers (*N* = 7,449)	Referral to substance abuse support: dichotomous “Yes/No” responses measured by youth self-report of the experience within the past 2 years
Rahamim and Mendes (2015)	Australia	Qualitative	Cross-sectional	Social care professionals (*n* = 9) Other professionals (*n* = 11)	Not applicable
Rebbe et al. (2017)	USA	Quantitative, SDA of existing study data*	Longitudinal, data collected from age 17 until the age 26	Care-leavers (*N* = 767)	Depression: currently meets criteria, using composite International Diagnostic Interview PTSD: same as above Drug dependence: same as above Access to treatment: question was added to the interview for the purposes of this study to assess whether participants had accessed treatment for conditions listed above
Rebbe et al. (2018)	USA	Quantitative, SDA of existing study data*	Longitudinal, data collected from age 17 until the age 26	Care-leavers (*N* = 732)	Eating disorders: interview question with dichotomous yes/no variable ADHD: same as above
Scheffers et al. (2019)	Canada	Quantitative, SDA of existing study data	Longitudinal, data collected around age 15 and around 19	Women care-leavers from residential care (*N* = 182)	Personality disorders: current symptoms using French adaptation of the PDQ4+
Sims-Schouten and Hayden (2017)	UK	Qualitative	Cross-sectional	Care-leavers (*N* = 22)	Not applicable
Sulimani-Aidan et al. (2021)	Israel	Quantitative, mixed sources	Cross-sectional	Care-leavers (*N* = 2,295)	Psychological distress: current symptoms using a 10-question screening scale of psychological distress
White et al. (2011)	USA	Quantitative, SDA of existing study data	Cross-sectional	Care-leavers (*N* = 542)	Mental health symptoms: met criteria for current diagnosis, using SCL-90-R PTSD: current symptoms, using PTSD Checklist for Civilians
Yates and Grey (2012)	USA	Quantitative	Cross-sectional	Care-leavers (*N* = 164)	Adaptation class: latent class analysis used to group young people in terms of their current level of adaptation, including symptoms of depression (depression subscale of the BSI, education achievement, employment, civic engagement, and self-esteem

*Note*. ADHD = attention-deficit/hyperactivity disorder; BSI = Brief Symptom Inventory; CASI-A = Comprehensive Addiction Severity Index for Adolescents; CES-D = Center for Epidemiologic Studies Depression Scale; GHQ = General Health Questionnaire; PDQ4+ = Personality Diagnostic Questionnaire 4+; PTSD = posttraumatic stress disorder; SCL-90-R = Symptom Checklist-90-R; SDA = secondary data analysis; TSC = Trauma Symptom Checklist.

* = uses the same sample as another included research study.

### Research Design

Four pieces of research were qualitative and the remaining 19 were quantitative in design. Of the quantitative research studies, one analyzed electronic administrative data (e.g., social care and health care records; *n* = 1), and three used a mixture of research data and administrative data. Two pairs of research papers conducted analyses on the same sample (Miller et al., 2017; Narendorf & McMillen, 2010; Rebbe et al., 2017, 2018). In all, 11 studies used longitudinal methods, and the remaining 12 used a cross-sectional design. Studies ranged from small sample sizes (at minimum, *N* = 11) to large population-based studies (at maximum, *N* = 913,207). Within the largest population-based study, the subsample of care-leavers was *N* = 7,449 (Prince et al., 2019). Characteristics of the included studies are presented in Table 1.

**Figure 2.**
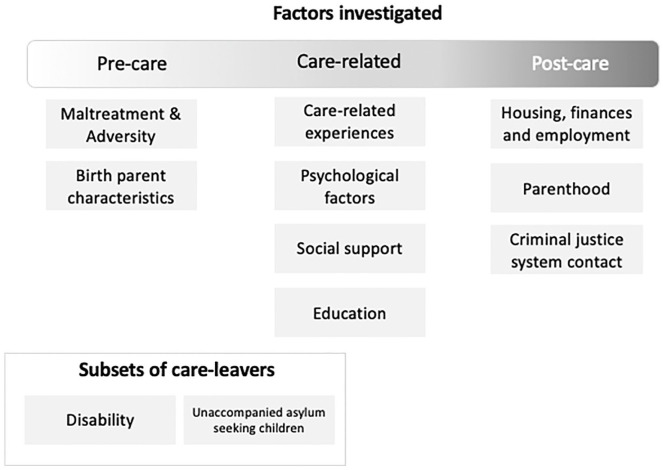
Factors investigated.

Mental health outcomes were measured in a variety of ways including semi-structured diagnostic clinical interviews of mental health, validated self-report symptom questionnaires, measures of suicidal behavior or ideation, or access to mental health, drug, or alcohol services use. Many used a combination of these methods, as summarized in Table 2. Associations with mental health outcomes spanned pre-care, in-care, and post-care experiences (see Figure 2).

**Table 2.** A Table of the Factors Investigated in the Included Studies and Findings.

Author(s)	Factors Tested										
	Pre-Care Factors		Care-Related Experiences	Psychological Factors	Social Support/Support Programs	Education	Post-Care Factors			Subsets of Care-leavers	Other
	Maltreatment and Adversity	Birth Parent Characteristics					Housing, Finances, and Employment	CJS Contact	Parenthood		
Berlin et al. (2011)						**✓**					
Cashmore and Paxman (2006)			~		**✓**						
Dixon (2008)			✘					**✓**			
Fowler et al. (2011)							**✓**				
Goldstein et al. (2013)	~			**✓**							
Grey et al. (2015)				~							
Gullo et al. (2021)										**✓**	
Kessler et al. (2008)			**✓**								
McCauley (2021)										✘	
Melkman (2017)	**✓**				~						
Miller et al. (2017)			~					**✓**	**✓**		
Munson et al. (2015)					N/A						
Narendorf and McMillen (2010)	~		✘	**✓**							
Newton et al. (2017)					N/A						
Prince et al. (2019)	✘		~		✘						
Rahamim and Mendes (2015)											N/A
Rebbe et al. (2017)	~										
Rebbe et al. (2018)	~										
Scheffers et al. (2019)	~										
Sims-Schouten and Hayden (2017)					N/A						
Sulimani-Aidan et al. (2021)		✘			~	✘	~		**✓**		
White et al. (2011)							**✓**				
Yates and Grey (2012)	✘		~		**✓**						

*Note*. CJS = criminal justice system; **✓** = Significant findings found; ✘ = No significant findings found; ~ = Mixed findings; N/A = qualitative research.

### Summary of research findings

Tables 3 and 4 provide an overview of the key findings of this review, as well as implications for practice, policy, and research.

**Table 3.** Critical Findings.

Summary of Critical Findings
• 23 studies exploring factors that influence the mental health of care-leavers were included • Evidence is mixed, sometimes contradictory, and subject to important limitations (e.g., over-reliance upon retrospective reporting and unvalidated measures) • The field benefits from several large-scale observational studies, though there are significant gaps in our knowledge around modifiable factors and mechanisms for associations • We found some consistent findings indicating that social support may be a protective factor for care-leaver mental health

**Table 4.** Implications.

Summary of Implications for Practice, Policy, and Research
• Methodological flaws should be addressed with future research, including more consistent use of validated mental health measures, longitudinal research with longer follow-up periods, and moving away from retrospective reporting • Future research should focus on modifiable processes, or theoretically driven mechanism research to identify processes that might support care-leavers toward better mental health • Social support may be a potential modifiable protective factor for mental health problems, but this requires further empirical investigation • This is a developing field with limited evidence as yet. Policy and practice changes should be well-evidenced meaning further research is needed before changes can be recommended

#### Birth parent characteristics

Only one research study investigated the impact of birth parent characteristics on mental health outcomes. With a sample of 2,295 care-leavers and using information within administrative records, Sulimani-Aidan et al. (2022) found that mothers’ marital status was unrelated to psychological distress of care-leavers, but parental use of welfare services and parents’ criminal convictions were related (β = .08, *p* < .02; β = .05, *p* < .05). However, once other factors were controlled for (see Table 2), these associations reached non-significance.

#### Childhood trauma and maltreatment

Maltreatment was measured in several ways: the “main reason for entering care” (*n* = 2); the severity of different types of maltreatment or adverse childhood experiences (*n* = 4), or a mixture of the two (*n* = 2). Of these eight studies, only two used standardized and validated measures of childhood maltreatment experiences. Across studies, two found no associations between maltreatment and mental health outcomes and the remaining, one found associations, and five found conflicting associations (e.g., associations between only certain types of maltreatment and certain mental health outcomes; often inconsistent between studies).

##### “Main reason” for entering care

Two studies used the “main reason for entering care” as a proxy for maltreatment history, and both found no associations with mental health outcomes. A large longitudinal study (*n* = 7,449), which used information from social care records, found no association between trauma-related “reasons for entering care” and referrals to substance misuse support at age 19 while accounting for other individual, pre-care, and in-care variables (Prince et al., 2019). A smaller-scale cross-sectional study of care-leavers in their early 20s (*N* = 127) examined associations between retrospective reports of the main reason for entering care (child abuse, neglect, parental substance abuse, and “other”), and profiles of adaptation (Yates & Grey, 2012). They identified four classes of adaptation: a maladapted group (high mental health and social difficulties; poor work or education outcomes); a resilient group (low mental health difficulties; low work or education difficulties); an internally resilient group (low mental health difficulties; high work or education difficulties); and an externally resilient group (high mental health difficulties; low work or education difficulties). They found no associations between the main reason for entering care and these classes of adaptation. They also found no associations between adaptive profiles and retrospectively reported maltreatment severity, measured using a validated verbally administrated inventory.

##### Pre-care maltreatment type and severity

Six research papers (based upon five research studies) examined associations between retrospectively reported pre-care maltreatment severity and mental health outcomes of care-leavers. One study of 354 care-leavers found a moderate positive correlation between retrospectively self-reported pre-care maltreatment and psychological distress around the age of 18 to 25 (*r*_pb_ = 0.48, *p* < .001; Melkman, 2017). The remaining five papers reported mixed or no evidence on the association between retrospectively reported pre-care maltreatment severity and mental health outcomes of care-leavers (Goldstein et al., 2013; Narendorf & McMillen, 2010; Rebbe et al., 2017, 2018; Scheffers et al., 2019).

For example, Narendorf and McMillen (2010) found that “severe” or “moderate” physical abuse (based on retrospective self-reported frequency) was related to higher odds of substance abuse disorder at the age of 19 but not 18 (odds ratio [OR]: 2.74, CI [1.05, 7.11]), when compared to low or no exposure (*N* = 325). Conversely, “severe” or “moderate” sexual abuse was related to less instances of Substance Abuse Disorders at age 19 with no associations at 18 (OR: 0.34 CI [.13, .92]). They found no associations between the severity of physical neglect and substance abuse disorders at 18 and 19. Similarly, physical abuse was unrelated to symptoms of depression with a sample of 93 young adults, but depression was related to the severity of sexual abuse, emotional abuse, and emotional neglect (*r* ranged from .24 to .35; Goldstein et al., 2013). Scheffers et al. (2019) examined the severity of retrospectively reported child maltreatment in relation to personality disorder symptoms in young adults who transitioned out of residential care (*N* = 182; e.g., schizoid, borderline, passive aggressive, depressive). When multiple forms of abuse were examined together, emotional abuse was the only factor associated with nearly all personality disorder symptom clusters (β ranged from 0.18 to 0.42, all *p* < .05).

Two papers which both used data from the Midwest Evaluation of Adult Functioning of Former Foster Youth study (Courtney et al., 2011) explored adversities both pre- and during care, using retrospective reporting (approximately 25 years; *N* ranged from 732 to 767; Rebbe et al., 2017, 2018). Three categories of early adversity were statistically derived: Complex Adversity (high incidents of nearly all types of adversity), Low Adversity (fewer types of adversity experienced), and Environmental Adversity (less interpersonal maltreatment and high rates of witnessing violence/being in a life-threatening accident). Across research papers, the complex adversity class had statistically more instances of depression, eating disorders, PTSD, and drug and alcohol abuse than the low adversity group (although differences were often marginal—e.g., 65% had depression compared to 60% in the low adversity class). The environmental class also had higher incidents of PTSD, drug and alcohol abuse compared to the low adversity group. No statistical differences between the complex and environmental adversity classes were found, and overall, no differences in groups were found in terms of rates of eating disorders.

#### Care-related experiences

Several studies investigated in-care or transition-related experiences in association with care-leaver mental health. The most frequently investigated care-related experiences were placement stability (primarily total number of placements) of which one out of four studies found significant associations, and age of exit from care, of which two out of four studies found associations.

##### Placement stability

A study with 106 care-leavers around a year after exiting care found that the severity of psychological distress was unrelated to the total number of placements (self-reported; Dixon, 2008). A large research study with 7,449 care-leavers also found no association between total number of placements (taken from administrative files) and substance abuse referrals at the age of 18 and 19 (Prince et al., 2019).

Using combined mental health and functioning outcomes, three further studies found no associations. Cashmore and Paxman (2006) used “adult functioning” as the outcome, whereby the participant scored “0” or “1” dependent on whether they had achieved success in various domains, which included mental health (e.g., absence of mental health problem, being in employment). Adult functioning 5 years after exiting care was better if the young person had fewer placements while in care, and if they had spent over 75% of their time in care within one placement (retrospectively self-reported; effect sizes not reported; Cashmore & Paxman, 2006). However, these associations did not survive adjustment for other factors (e.g., social support; see Table 2 for other factors investigated). In addition, a study with 164 care-leavers found both the total number of placements (self-reported) were unrelated to classes of adaptation around 12 months after exiting care (Yates & Grey, 2012).

##### Age of exit from care

Several research studies examined the age of exit from care (ranging from 16 to 19 years old across studies) and mental health post-leaving care, with no consistent findings. Dixon (2008) found no association between psychological distress 1 year after exiting care, and age of exit from care (75% left before the age of 18; *N* = 106). This was also the case for Narendorf and McMillen (2010) who found no association between substance abuse disorders and exiting before or after the age of 18 (*N* = 325). By contrast, in their study of 7,449 care-leavers, Prince et al. (2019) found that exiting care at the age of 19 (as opposed to before this) was associated with increased odds of substance abuse referrals (OR: 1.49, *p* < .01). Finally, in a study with 164 young people, the group which was classed as Externally Resilient had the highest age of exit from care (*M* = 18.56, *SD* = 0.65; *N* = 164), and this was statistically higher than Maladapted and Internally Resilient groups (*F* = 3.45 (3, 160), *p* = .02; Yates & Grey, 2012).

##### Foster care programs

Using an observational design, Kessler et al. (2008) evaluated the effects of a privately funded foster care program for youth aged 14+ (Casey Family Program, *n* = 111) on youth mental and physical health, compared to an unmatched group of young people who grew up in state foster care (*n* = 368). The Casey Family Program was characterized by staff members with higher levels of education, higher salaries, and lower caseloads. Youth in the program had access to a wide range of support services (e.g., mental health counseling, tutoring, and summer camps), stayed in care until an older age, had fewer placement moves, experienced fewer adverse events in care, and were less likely to experience a reunification failure. Casey alumni had a significantly lower 12-month prevalence of depression (11% vs. 23%), anxiety (29% vs. 43%), and substance use disorders (5% vs. 11%) in young adulthood (approximately 25 years old) than the control sample.

##### Other placement factors

Cashmore and Paxman (2006) found that continuity of living situation across the transition out of care (i.e., continuing to live with foster parents) was associated with better “functioning” after leaving care (β = 0.396, *p* < .05). Prince et al. (2019) found that having a “runaway” status and being in a residential home just before leaving care was related to substance abuse referrals at 19, but not 18 (OR: 1.77, 95% CI [1.26–2.50]; OR: 1.50, 95% CI [1.22–2.50]). Yates and Grey (2012) explored the influence of being placed with siblings, finding that being placed with versus without siblings differed among adaptation classes, though post hoc comparisons failed to reach significance (*F*(3,160) = 2.91, *p* = 0.36).

In a wider exploration of transitions out of care, a longitudinal study with more than 400 recent care-leavers, statistically derived several classes based on preparedness to leave care at age 17 (Miller et al., 2017). These were the Moderate Problems class (more likely to have been arrested, repeat a grade, and experience homelessness); the Resilient class (low instances of arrests, repeating grades, one living situation in the past year); the Multiple Problems class (characterized by homelessness, >4 living situations in the past year and living in a residential home); and the Pregnancy History class (experienced pregnancy and >4 living situations in past year). At the age of 19, the Multiple Problems class was the most likely to meet the criteria for PTSD and substance use disorder, and both the Multiple Problems and Pregnancy History class had higher rates of depression and depression symptoms than the other classes.

#### Individual psychological factors

Two research studies investigated psychological factors in relation to mental health problems in young adulthood, and both found significant associations.

Goldstein et al. (2013) examined “internal resilience” using a validated measure of resilience with a sample of 93 young adult care-leavers. Goldstein et al. (2013) found that resilience was negatively associated with symptoms of depression in adulthood, including when demographics and retrospectively reported maltreatment were entered into the model (*B* = −0.23, *p* < .05). Grey et al. (2015) examined current coping styles in the context of resource availability. In a sample of recently transitioned care-leavers (*N* = 172), they studied the association between an active, support-seeking, or cognitive reframing coping style, and resource availability in terms of housing stability, perceived social support, and education level, respectively. They found a significant interaction between active coping style and housing stability, whereby depression was higher for those with an active coping style, who also had unstable housing (interaction: β = −0.15, *p* < .05). An active coping style, and housing stability was unrelated to anxiety symptoms. A support-seeking coping style, in the context of low perceived social support, was related to both higher symptoms of depression (interaction: β = −0.3, *p* < .001) and anxiety (interaction: β = −0.3, *p* < .001). Finally, those who had a cognitive reframing coping style, and a lower education level, had higher symptoms of depression (interaction: β = −0.17, *p* = .028). There was no interaction effect with anxiety, but cognitive reframing was significantly related to anxiety, whereby higher anxiety was associated with cognitive reframing (β = 0.24, *p* < .05).

#### Mental health

One research study examined the influence of childhood mental health problems on mental health outcomes in care-leavers. Specifically, in a sample of 325 care-leavers Narendorf and McMillen (2010) found that having a conduct disorder diagnosis in childhood was related to a substance use disorder at ages of 18 and 19 (OR = 3.33, 95% CI [1.34, 8.30]; OR = 3.12, 95% CI [1.24–8.31]), controlling for demographics, retrospectively reported maltreatment history, peer substance use, and age of exit.

#### Social support

Social support was examined in six studies, two of which used validated measures of social support, while the rest used unvalidated or single-item measures. Of the three studies that examined relations between pre-care social support and post-care mental health outcomes, two found significant associations. All three studies which examined current perceived social support found associations with concurrent mental health outcomes.

##### Retrospectively reported pre-care perceived social support

Prince et al. (2019) found no association between having a “connection to a caring adult” at the age of 17 years (single item) and substance abuse referrals at ages 18 and 19 (*n* = 7,499). Similarly, with a smaller sample of 2,295 care-leavers, having caring residential support staff while in care (retrospectively reported using an adapted questionnaire) was unrelated to psychological distress. In contrast to Prince et al. (2019), Sulimani-Aidan et al. (2022) found that reports of low peer support while in care (retrospectively reported using a standardized questionnaire) was negatively related to psychological distress at the age of 27 (β = −.11, *p* < .01), even adjusting for key personal characteristics, qualities of the in-care and post-care environment, and life satisfaction across multiple domains (Sulimani-Aidan et al., 2022; see Table 2). In line with this finding, Cashmore and Paxman (2006) retrospectively measured “perceived emotional security” while in care and found it was associated with better “adult functioning” after leaving care (β = .44, *p* < .001; *n* = 47).

##### Current post-care social support

Cashmore and Paxman (2006) also measured the current number of positive sources of support they had 4 to 5 years after exiting care (unvalidated measures). Greater sources of social support after leaving care was related to better “adult functioning” after leaving care (β = .33, *p* < .01; β = .44). Yates and Grey (2012) replicated these findings with 164 care-leavers, using validated measures of current perceived social support and peer attachment, whereby care-leavers who scored highly (indicating higher levels of support and attachment), were more likely to have a Resilient profile (fewer depression symptoms and better adult functioning).

In their cross-sectional study of 345 care-leavers, both structural features (e.g., size, frequency of contact) and subjective features (adequacy and satisfaction) of emotional, practical, and informational support networks were negatively related to psychological distress (Melkman, 2017; *r*_pb_ ranged from −.11 to −.37). Mediation models tested the pathways between retrospectively reported childhood adversity, social network features, and psychological distress. For both practical and informational support networks, greater retrospectively reported childhood adversity was related to lower network adequacy, and partially mediated the association with psychological distress. All other mediation pathways with psychological distress were nonsignificant.

Finally, using qualitative research methods, Munson et al. (2015) explored supportive relationships during the transition out of care for recent care-leavers with mental health problems. Characteristics that were deemed important across the sample (*N* = 59) were as follows: consistency and availability of support; feeling loved and connected; having a shared understanding of experiences (e.g., trauma, mental health); open communication; reciprocity of support and finally, unconditional positive regard. The nature of support provided by key adults spanned encouragement, informational, and practical support.

##### Support programs

Two large-scale quantitative pieces of research examined whether accessing skills-based programs was related to mental health (e.g., budget and home management, skills). Neither Sulimani-Aidan et al. (2022) nor Prince et al. (2019) found that accessing life-skills programs was related to positive mental health outcomes (substance abuse referrals at 19, and psychological distress around the age of 27), once pre-care and in-care variables were also entered into the model (*N*s ranged from 2,295 to 7,449).

Two UK-based qualitative studies investigated the utility of a skill-based program (Sims-Schouten & Hayden, 2017; *N* *=* 22) and a mentorship program (Newton et al., 2017; *N* = 11) in improving the outcomes of care-leavers (including mental health). Sims-Schouten and Hayden (2017) found that young people held conflicting views of themselves as being vulnerable and having very complex needs while preferring independence, and feeling pressure to gain “resilience” to fit into society. They argued that a better understanding of how care-leavers conceptualize mental health was required to develop life-skills programs which target this. For Newton et al. (2017), themes emerged around the pressing need for transitional support; the importance of utilizing people in their existing social network; and the need to feel valued and to have open-ended support. They concluded that support programs should aim to bolster existing social connections, as opposed to forging new time-limited and formal relationships.

#### Education

Two studies examined education in relation to adult mental health, and the findings were mixed. Berlin et al. (2011) investigated the association between achievement in school and rates of suicide attempts using birth-cohort data. They compared outcomes between care-leavers (*n* = 5,224), majority population peers (*n* = 900,322), national adoptees (*n* = 1,205), and peers who had received in-home interventions before age 13 (*n* = 6,455). Care-leavers had a seven-fold increased risk for suicide attempts above the majority population group (RR = 6.94, 95% CI [6.14,7.84]), but after adjusting for sex, year of birth, maternal education, birth parents’ substance abuse, and psychiatric care, and average grade point in school at ages 15–16, suicide rates reduced by three-fold (RR = 2.28, 95% CI [1.97, 2.65]). Conversely, Sulimani-Aidan et al. (2022) found that educational achievements by the age of 18 were unrelated to psychological distress around the age of 27 (*N* = 2,295).

#### Housing, employment, and finances

Four research studies examined mental health outcomes in relation to post-care housing, employment, and finance variables, most of which found associations between the stability of housing, employment, and finances and mental health problems in care-leavers.

With 265 recent care-leavers, Fowler et al. (2011) derived three profiles of “transitions out of care” which represented stability of housing, employment, and education spanning 2 years after exiting care: Stable-Engaged class (stability across housing, employment, and education; Stable-Disengaged (stable housing but changes in education and employment status) and Instable-Disengaged (homelessness, did not complete education or employment). Overall, the stable-engaged class and stable-disengaged group had similar levels of emotional distress 2 years after exiting care, but the instable-disengaged class reported greater emotional distress than the stable-engaged class (OR: 2.14, 95% CI [1.16–3.94]). Similarly, White et al. (2011) found that those experiencing homelessness in the immediate years after leaving care were nearly two times more likely to have a probable mental health disorder (59% vs. 32%) and were three times more likely to have PTSD (14% vs. 4%) around 25 years old, than care-leavers who did not experience homelessness (*N* = 542).

Sulimani-Aidan et al. (2022) created a combined “satisfaction with finances, employment, and housing” variable, and with a sample of 2,295 care-leavers aged 27, and subjective satisfaction was negatively related to psychological distress (*ß* = −.17, *p* < .01). Greater levels of material deprivation (validated self-report questionnaire) were also related to greater psychological distress (*ß* = .19; *p* < .01), including adjustment for key personal characteristics, in-care, and post-care experiences. However, monthly earnings and the use of welfare services (e.g., income support) were not associated with psychological distress, once these other variables were controlled for. This finding was replicated by Prince et al. (2019), whereby housing burden (indicated by average percentage income spent on rent) was related to substance abuse problems at 19 (*N* = 7,449).

#### Criminal justice system contact

Across two studies, juvenile and post-care offending was inconsistently associated with poorer mental health. With a sample of 404 care-leavers, Miller et al. (2017) found that the latent classes characterized by contact with youth juvenile services (Moderate Problems class) were more likely to have a substance abuse disorder than the Resilient or Pregnancy History class, but rates of depression, PTSD, and anti-social personality disorder were comparable. With a sample of 106 care-leavers, Dixon (2008) found a positive association between offending in the immediate period after leaving care, and psychological distress 12 months after leaving care.

#### Parenthood

Two research studies examined parenthood and its association with mental health. In research with more than 400 care-leavers aged 19, the adaptive class which had high instances of parenthood (the Pregnancy History class), had the least instances of substance use disorder and PTSD, but the highest levels of depression (Miller et al., 2017). Sulimani-Aidan et al. (2022) found that those who were parents by the by the age of 27, had lower levels of psychological distress (*N* = 2,295; β = −.05, *p* < .01).

#### Subsets of care-experienced people

Two pieces of research examined mental health within subsets of the care-experienced population. McCauley (2021) explored whether disability status (taken from administrative data) relates to substance abuse (*N* = 5,221). Having an emotional/mental, sensory/physical disability, or “other” disability was unrelated substance abuse. Gullo et al. (2021) examined the mental health outcomes of unaccompanied young migrants (*n* = 68), compared to an unmatched group of care-leavers aged 18 to 25 (*n* = 73). Unaccompanied young migrants had less suicide ideation, though suicidal ideation was low across the entire sample (nonsignificant differences for suicide attempts; φ_c_ = 0.23; *p* < .01; φ_c_ = 0.15, *p* > .05).

#### Other

Finally, a piece of qualitative research by Rahamim and Mendes (2016) aimed to broadly investigate the support needs of care-leavers with mental health problems, as they transition out of care. They interviewed 19 social care professionals working in leaving care teams, and 11 other professionals who often work with care-leavers (e.g., youth justice, mental health, or homelessness workers). Professionals perceived the longstanding influence of early experiences of trauma in before entering care, and the challenges arising from being in care (e.g., frequent placement moves) as key to mental health. Various policy and practice challenges were also identified as influencing mental well-being of care-leavers, including a focus upon crisis management and practical functioning, unfit support options from mental health services, and the high turnover of social care staff with minimal qualification requirements for working in leaving care teams. Social care practices which were deemed protective for care-leavers mental health were as follows: directly supporting mental health needs; better multi-agency communication; outreach services to reduce isolation; training leaving care teams in mental health; establishing stable caring relationships; and finally, providing support beyond age thresholds.

## Discussion

We aimed to systematically scope the international literature related to factors that impact the mental health of care-leavers. In all, 23 studies were included, which were all from high-income western countries. These studies investigated the following: maltreatment, care-related experiences, psychological factors, social support, education and adult functioning (e.g., housing, finances, employment). This review highlights the heterogeneity of research in this area, utilizing quantitative and qualitative methods, cross-sectional and longitudinal designs, and spanning psychological and social care fields. There was very little consistent evidence across studies for any particular associations with care-leaver mental health, including studies using similar methodologies. However, this may be due to methodological flaws. For example, many studies did not use validated or standardized measures and relied upon retrospective reporting (see Table 1).

Several pieces of the included research examined maltreatment in relation to mental health outcomes of care-leavers, but there was little consistent evidence that pre-care maltreatment was robustly associated with mental health. Some studies found no associations, while others found some for specific ages but not others (e.g., 19 but not 18 years old), or for specific types or severity of maltreatment. No two studies measured or analyzed associations between maltreatment and mental health outcomes in the same way, meaning it is difficult to contrast findings. In the general population, experiencing childhood maltreatment (as opposed to not) is a transdiagnostic risk factor for virtually all commonly occurring forms of mental health difficulties across the lifespan, with little meaningful variation in the strength of associations across disorders (Kessler et al., 2010; McLaughlin et al., 2020). When comparing samples of maltreated versus non-maltreated individuals, research consistently shows higher rates of mental health difficulties (e.g., Hughes et al., 2017; Xiao et al., 2022), and complex trauma (frequently experiencing multiple types of maltreatment) is associated with greater psychological problems than single-event trauma (Finkelhor et al., 2011). In that sense, maltreatment is a potent risk factor for mental health. However, in this review, the majority of samples had experienced some form of maltreatment and high rates of other adversities. The mixed evidence suggests caution is warranted in terms of using “extent of maltreatment” as a potential way to gauge risk of later outcomes, or to as a proxy of need for support for care-leavers. This is important given qualitative research also reviewed here found that professionals consider maltreatment history as key to mental health needs in care-leavers.

There are many challenges to measuring maltreatment which make it difficult to draw conclusions and may explain the inconsistency of results. Nearly all studies relied upon retrospective self-reporting maltreatment reported years after events took place. Retrospective reporting may be influenced by their current well-being, making causality impossible to establish (Danese et al., 2021). Included administrative research studies often used court records as sources of information, which avoided issues with retrospective reporting, but are likely to be an insensitive measure of actual maltreatment exposure (Asmussen et al., 2020; Francis et al., 2023). There has also been much debate around the utility of categorizing types of maltreatment, given subtypes commonly co-occur (Warmingham et al., 2019). Attributing mental health outcomes to specific maltreatment subtypes ignores the individual’s cumulative experience of maltreatment. Some of the studies reviewed here did find positive associations between subtypes and outcomes, but there was little agreement between studies about which “types” were associated with outcomes. Broadly speaking, this points to methodological issues within the field, around measuring and researching maltreatment. Of note, only two research papers, which used the same sample, considered the impact of adverse childhood experiences after entering care. Given the documented risk of revictimization across the lifespan for people who experience maltreatment in childhood, this remains an important area of research (Euser et al., 2016; Katz et al., 2020).

Across studies, there was limited evidence of the impact of in-care experiences upon mental health after leaving care (e.g., age of entry, age of exit). There was also little evidence that placement stability (total number of placements) was associated with later mental health, which was unexpected given the majority of included samples in this review were young adults, meaning that many had left care relatively recently. Reviews of the child and adolescent literature indicate a well-evidenced association between placement instability and mental health while in care, with this likely reflecting a bi-directional association (Engler et al., 2022; Konijn et al., 2019; Lee & Holmes, 2021). In this review, placement stability was examined alongside a myriad of other factors, meaning its effect is masked by other factors which are more salient in the out-of-care context (e.g., total placements may also not be sensitive to the quality of the placement or relationship quality with the caregiver). Social support is rarely controlled for in the childhood and adolescence field (Lehmann et al., 2013; Okpych & Courtney, 2018). What is clear, is that stability in the immediate period before leaving care may be indicative of greater mental health needs after leaving care. In particular, homelessness and running away may indicate pervasive mental health needs. This has important implications for the allocation of resources for social care departments as young people reach the age of exit from care.

There were mixed findings in relation to the age of exit from care and mental health outcomes. It is difficult to draw implications from this review given that in all studies the eldest “exit age” was 19, and this was homogeneous across the samples (17–19 years). It is also challenging to draw implications from this research, given the circumstances around the exit are not well understood, and there are likely to be many unmeasured confounding factors. For example, some young people may leave care later because they require more prolonged support. It is generally understood that continued support past the age of 18 has the potential to be protective for mental health and other life outcomes. It appears that as yet, continued care beyond the age of 18 has not been investigated in terms of mental health within the scientific literature. Providing continued care will have economic implications for social care services, but may protective in terms health and employment outcomes for example, meaning there may be potential savings in terms of service utilization (e.g., hospital visits, welfare services). Research is needed to understand what continued support should involve, and to test whether this could improve mental health outcomes after transitioning out of care.

Very few research studies examined associations between psychological factors and mental health outcomes in care-leavers. The research studies which did so investigated internal resilience and coping styles, which may be considered broadly analogous to emotion regulation. Both internal resilience and coping styles were found to be associated with fewer depression and anxiety symptoms, including when childhood maltreatment was controlled for in analyses. This corroborates findings within the general population, which indicates strong ties between maltreatment and emotion dysregulation, and mental health problems (e.g., Gruhn & Compas, 2020; Visted et al., 2018; Weiss et al., 2022). Research within the trauma field indicate strong ties between psychological processes which are impacted by trauma (e.g., threat appraisals) and psychological outcomes (e.g., McCrory et al., 2022; McLaughlin et al., 2020). This could be a changeable target for improving mental health care-leavers, but given the methodological flaws of the included studies, further work is needed on this.

Of note, the association between childhood mental health problems and adult difficulties was largely unexplored. This remains an important area of investigation in this population, given the age of onset of mental health problems in the general population is usually within childhood or adolescence, and that subclinical symptoms in childhood are associated with later mental health problems (Copeland et al., 2015; Kessler et al., 2007). It is likely that care-leavers who were experiencing problems in young adulthood were during childhood too, yet trajectories of mental health difficulties into adulthood is not yet understood within this population.

Several research studies investigated social support, and findings were largely uniform, with better perceived social support being associated with better mental health. This was the case when studies used simple single-item measures as well as standardized self-report measures, and in studies which controlled for individual, pre-care, and in-care variables. However, like with maltreatment measures, there was a reliance on retrospective reporting of in-care perceived social support, raising issues of single-informant bias and meaning causality cannot be inferred. The perception of their earlier support may be influenced by their current well-being. Yet, qualitative literature builds upon these findings, indicating that young people exiting care with mental health problems value relationships which feel reciprocal and that are long lasting, and this may be protective for mental health (Munson et al., 2015). Within the wider literature, social support is considered protective of mental health problems transdiagnostically, especially when trauma is experienced in childhood (Trickey et al., 2012). Several psychological models link social support to mental health (e.g., neurocognitive models McCrory et al., 2022); maladaptive cognitions (e.g., Lanctôt, 2020); stress-buffering model (Cohen & Wills, 1985), but these models remain untested with the care-leaver population.

Conversely, research on the utility of support programs was very limited. We did not identify any trials, randomized and controlled or otherwise, which tested support programs in terms of mental health outcomes of care-leavers. This requires more direct investigation, given that internationally, skills-based support programs are commonly offered to care-leavers (Strahl et al., 2021). Existing qualitative literature on this topic has the potential to provide insight into the formation of support programs. For example, care-leavers expressed the importance of nurturing existing relationships, as well as enabling care-leavers to feel more comfortable reaching out for support (Newton et al., 2017).

The remaining literature examined factors such as education, employment, finances, and housing, and as expected found that those who were functioning well (e.g., completed compulsory education, had stable employment and housing) also had better mental health after leaving care. This relationship is likely to be bi-directional and might highlight the wide-reaching impact of unresolved mental health problems, and the importance of addressing these. In a recent report, leveling up emotional, financial, and educational support offered to care-levers was identified as a key area toward enhancing the well-being of care-leavers by >1,000 care-leavers in the UK (Briheim-Crookall et al., 2020). This indicates the importance of addressing mental health needs alongside education, employment, and housing support, or vice versa.

Only one research study examined disability status, which is surprising given the high prevalence of disability in the care-leaver population (Briheim-Crookall et al., 2020), and the link between disability, neurodiversity, and mental health problems (e.g., Adamis et al., 2023). Only one small-scale research study examined the mental health outcomes of unaccompanied young migrants, making it difficult to draw inferences on how to support this group of care-leavers. Worldwide conflicts and climate change have already increased the number of displaced people seeking asylum internationally, and this is only set to increase over the next few decades (e.g., Geisler & Currens, 2017). This is an area which will require further investigation internationally, to improve our understanding of how to support the mental health of these populations.

### Gaps and opportunities for growth

The field benefits from several large-scale pieces of research using large cohorts of young people, and some of these simultaneously investigate many factors. Just over half of the included studies used longitudinal designs, but some of the included studies still involved retrospective reporting. In addition, studies included in this review had short follow-up periods, of only a few years during early adulthood. It should be noted that some studies included had longer follow-up periods (e.g., Kessler et al., 2008), but research using these timepoints have not yet been published in peer-reviewed journals so could not be included here. These methodological flaws make it challenging to infer whether the associations reported are causal. Large longitudinal birth-cohort studies may address these methodological problems, but require significant resources, especially given the challenges of retaining transient populations (such as care-experienced people) within research.

Within some of the largest included studies, mental health outcomes were grouped alongside other functional outcomes (e.g., employment, housing, and mental health; see Table 1), meaning that it is difficult to discern which factors were the most influential for mental health specifically. Similarly, several studies used access to services (e.g., substance dependency services) as a proxy for mental health problems. What the core mental health difficulties may have been (alongside potential addiction issues) are unknown but are likely to be diverse in this group. It is also worth noting that there is growing evidence indicating barriers to mental health support for care-experienced young people, meaning outcomes focusing on service-access alone are unlikely to be representative of the overall group of young people (e.g., Phillips et al., 2023).

Many of the factors investigated were basic and often centered around general descriptions of the in-care journey (e.g., age of entry to care, number of placements). In addition, many of them were static and cannot be changed once they have occurred. For example, experiences of placement instability cannot be changed once they have left care. Few studies identified processes which might help care-leavers flourish despite these experiences, and mechanisms that drive these associations were under-explored. Social care systems have limited resources, meaning that research should focus on personal or interpersonal modifiable factors that can be nurtured (e.g., social support, psychological factors). There should also be ongoing efforts to understand effective interventions at the individual, social, and practice levels, which might improve mental health outcomes for this group. Examples might be interventions for the young person, for caregivers, within education settings, psychoeducation for professionals, and the use of evidence-based practice, to name a few examples.

To move the field forward, it may be beneficial to focus on well-designed cohort studies using validated measures, or experimental intervention research studies using matched samples. As a next step, we should draw upon psychological theory, and what is already understood about mechanisms in the mental health literature, but test this within the care-leaver context. For example, contemporary models of risk and resilience, or multi-system developmental models (e.g., Bronfenbrenner & Morris, 2006; McCrory & Viding, 2015; McLaughlin et al., 2020).

### Limitations of the review

Although this review has many strengths, highlighting research findings and gaps in an emerging field of research, it should be viewed in light of limitations. First, it is important to note that the study designs used mean causality can largely not be inferred due to methodological limitations. Second, prior to this decade, it was uncommon to differentiate between people who have some experience of being in care, and young people who age out of care (care-leavers). Where possible, we contacted authors for clarification on the care-status of their participants, but in many instances, this level of detail was not gathered by researchers at the time of undertaking the research. We also screened several studies that examined the impact of poor mental health on life outcomes, as our review was focused specifically on mental health as the outcome. For this reason, research which investigated related but distinct concepts (such as well-being, life satisfaction, or quality of life) was excluded. Third, this is an international review, and while we aimed to include search terms which are used internationally, it may be that we missed key terms and therefore research. Finally, we only screened academic literature, which may have led to the exclusion of rich gray literature, such as the Brightspots program (e.g., Briheim-Crookall et al., 2020).

### Summary

This review mapped the current state of peer-reviewed evidence investigating risk and protective factors associated with the mental health of care-leavers. Broadly speaking, findings were mixed and inconsistent across research studies, possibly due to methodological problems that should be addressed with future research. There was little consistent evidence that pre-care maltreatment was associated with the mental health of care-leavers, but generally good evidence (albeit with some methodological limitations) that social support, emotion regulation skills, and stability in the years approaching “aging-out” of care, may be important factors associated with mental health in the years following leaving care. The field benefits from several large-scale longitudinal research studies, but oftentimes this only spans a few years, with follow-ups that end in early adulthood, and limitations in the measures/variables available. To move the field forward, researchers should focus on mechanism-based research, or quasi-experimental studies, to identify changeable factors that might promote the mental health of care-leavers, as well as the continued use of existing administrative datasets to understand predictors of longer-term outcomes for this group.

## Supplemental Material

sj-docx-1-tva-10.1177_15248380231196107 – Supplemental material for A Scoping Review of Factors Associated With the Mental Health of Young People Who Have “Aged Out” of the Child Welfare System Supplemental material, sj-docx-1-tva-10.1177_15248380231196107 for A Scoping Review of Factors Associated With the Mental Health of Young People Who Have “Aged Out” of the Child Welfare System by Alice R. Phillips, Sarah L. Halligan, Iris Lavi, , John A. A. Macleod, Susan Robinson, David Wilkins and Rachel M. Hiller in Trauma, Violence, & Abuse
