# Supplementary material for: A Scoping Review of Factors Associated With the Mental Health of Young People Who Have “Aged Out” of the Child Welfare System
Source: Trauma Violence Abuse. 2023 Sep 30;25(3):1780–98. doi: 10.1177/15248380231196107 (PMC11155215; doi:10.1177/15248380231196107)
Supplement: sj-docx-1-tva-10.1177_15248380231196107 – Supplemental material for A Scoping Review of Factors Associated With the Mental Health of Young People Who Have “Aged Out” of the Child Welfare System [file sj-docx-1-tva-10.1177_15248380231196107.docx]

**Appendix 1**

**Search terms**

(“care leaver*” OR {aging out} OR {aged out} OR {foster alumni} OR {foster youth} OR ((leaving OR former OR emancipat* OR agi* OR transition) AND ({child welfare} OR {out of home} OR {looked after} OR {foster child} OR {foster youth} OR {foster care} OR {kinship care} OR {residential care} OR {state care} OR {out of care}))

AND

{mental health} OR {mental wellbeing} OR psychopathology OR psychiatr* OR “emotion* problems” OR “emotion* distress” OR “psych* distress” OR externali* OR internali* OR depression OR depressed OR anxi* OR suicid* OR “self harm” OR conduct OR oppositional OR psychosis OR (psych* W/4 disorder) OR (psych* W/4 problem) OR wellbeing OR well-being

**Appendix 2**

**Changes from pre-published protocol**

We made the following edits to the pre-published protocol. We originally proposed to look at the evidence for the ‘transition’ out of care. However, the pilot showed this initial conceptualisation was too narrow. Thus, the review was broadened to focus on care-leavers, rather than specifying the focus on their transition out of care. Second, we changed our abstract screening process to be more thorough in terms of the percentage of articles screened as part of inter-rater reliability. Finally, we had originally proposed to scope the international grey literature. However, it proved unfeasible to scope and review the grey literature world-wide by including, for example, reports by country specific public bodies and charitable organisations. Due to concerns about the potential bias that could be introduced by a selective approach to the grey literature, compared to the systematic nature of the academic review, we ultimately decided to focus only on the academic literature.
